# A well-preserved partial scapula from Japan and the reconstruction of the triosseal canal of plotopterids

**DOI:** 10.7717/peerj.5391

**Published:** 2018-08-25

**Authors:** Tatsuro Ando, Keisaku Fukata

**Affiliations:** 1 Ashoro Museum of Paleontology, Ashoro, Hokkaido, Japan; 2 Yamaguchi Prefectural Government, Yamaguchi City, Yamaguchi Prefecture, Japan

**Keywords:** Triosseal canal, Oligocene, Scapula, Plotopteridae, Penguins, Wing-propelled diving birds

## Abstract

The discovery of a well-preserved cranial end of a plotopterid scapula from the Early Oligocene Jinnobaru Formation in southwestern Japan has provided a fine example of its bone structure and has enabled the reconstruction of the triosseal canal (canalis triosseus) of the unique extinct penguin-like bird. It is believed that plotopterids performed penguin-like underwater propulsion using wings that were similar to those of penguins. Until this discovery, the lack of well-preserved plotopterid scapulae hindered reconstruction of the canalis triosseus, which is an important structure for the wing-upstroke. We reconstructed a composite model of the canalis triosseus based on the new scapula. The reconstructed size of the canal is as large as that in Emperor Penguins (*Aptenodytes forsteri*), suggesting that the bird had a large and powerful m. supracoracoideus, which is the essential muscle for the powered upstroke required for wing-propelled diving. Plotopterids likely have had the same functional requirement as penguins, the powerful wing-upstroke in the water. They must have also been capable swimmers. This scapula accounts for the structural difference between plotopterids and penguins in terms of the canalis triosseus. The large canalis triosseus of plotopterids was composed of the elongated acromion of the scapula, while penguins have a long processus acromialis claviculae for the same function.

## Introduction

**Figure 1:**
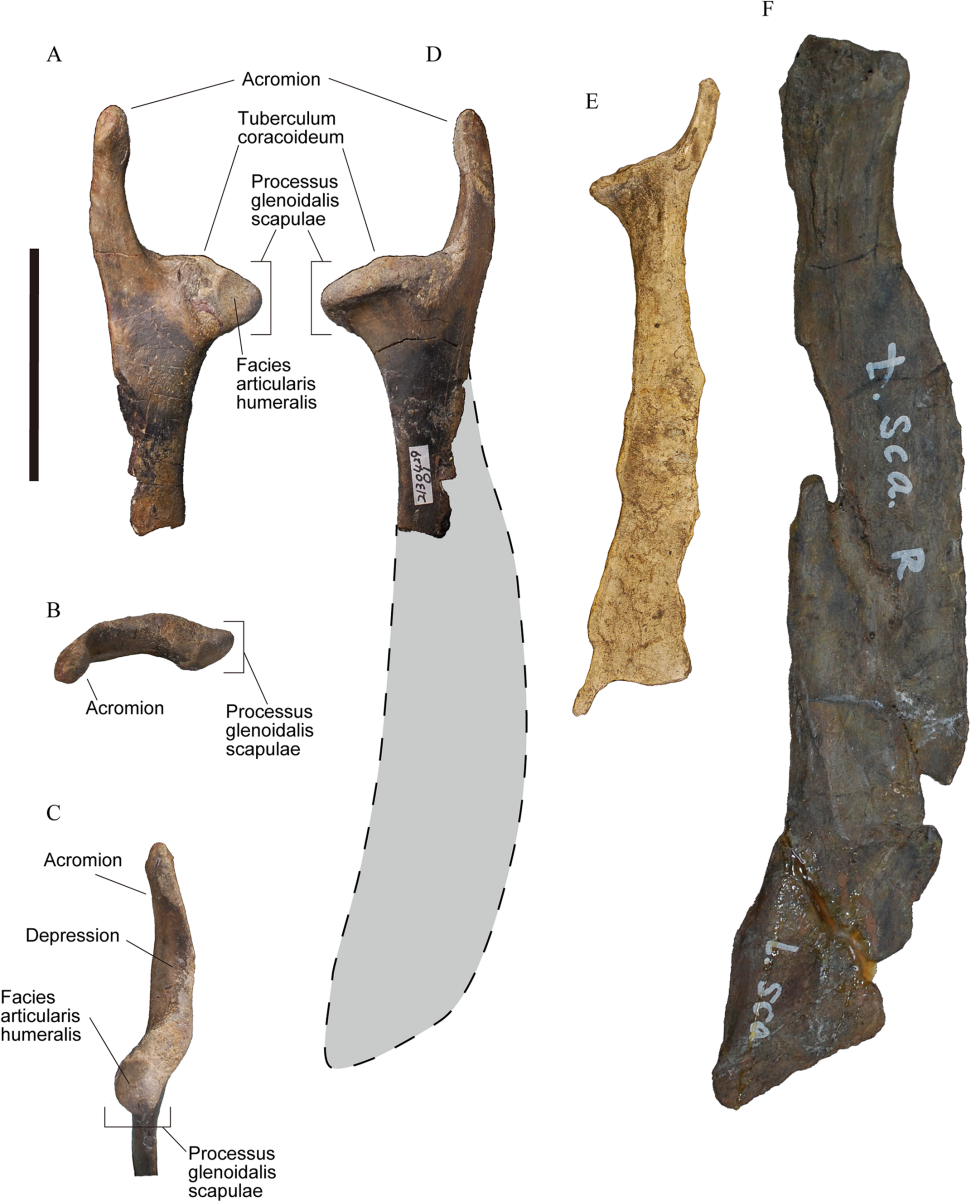
Right scapulae of plotopterids. (A)–(D) YM-G-100206. (E) Cast of *Tonsala hildegardae*, USNM 256518. (F) Cast of *Hokkaidornis abashiriensis*, AMP 44. (A) Lateral view. (B) Proximal view. (C) Cranioventral view. (D)–(F) Medial view with reconstructed outline (only D). Scale bar = 50 mm.

Plotopteridae is an extinct bird family, known as the northern counterpart of penguins, which were widely distributed in the North Pacific (Howard, 1969; Olson & Hasegawa, 1979; Mayr & Goedert, 2016). Their fossil records range from the Late Eocene to the Middle Miocene. Several common structures shared convergently with penguins and auks in the wing and shoulder girdle elements, such as short, dorsoventrally compressed wing bones, or a long processus acrocoracoideus, indicate that plotopterids were flightless, wing-propelled diving birds. However, detailed discussions on their function and ecology have been limited (Howard, 1969; Olson, 1980; Ando & Fordyce, 2014). A total of 11 species in eight genera of plotopterids have been described, but the scapulae, especially the cranial parts, are not fully described because of incomplete preservation. A nearly whole scapula is known only in *Tonsala hildegardae* (USNM 256518), but the fine structure is lost because of the poor surface preservation (Olson, 1980). In a *Hokkaidornis abashiriensis* specimen (AMP 44), the cranial scapula (Fig. 1) is damaged (Sakurai, Kimura & Katoh, 2008) although it can indicate the whole length of the scapula without an acromion. A poorly preserved partial left scapula was mentioned in *Copepteryx hexeris* (KMNH VP 200,006), but was neither described nor shown (Olson & Hasegawa, 1996). In *Klallamornis abyssa*, only the caudal portion is preserved (Mayr & Goedert, 2016).

The canalis triosseus is a uniquely derived structure of ornithothoracine birds and one of the key features that supports their powerful flight (Olson & Feduccia, 1979). The canal acts as a pulley to transmit the power from the m. supracoracoideus to the dorsal surface of the humerus. The tendon of the m. supracoracoideus runs through the canal, changing the direction of the force and lifts the wing in the upstroke. The canalis triosseus is formed by the processus acrocoracoideus of the coracoids, the acromion of the scapula, and the processus acromialis claviculae (Baumel & Witmer, 1993). The primary wing-upstroke muscle, the m. supracoracoideus, and the canalis triosseus are essential to the powered flight of volant birds, and the development of these structures is strongly linked to the evolution of modern birds (Olson & Feduccia, 1979; Poore, Sánchez-Haiman & Goslow, 1997; Mayr, 2017).

Wing-propelled diving birds tend to have a m. supracoracoideus of greater mass than non-diving birds since they need to overcome the drag on the wing in the water (Hui, 1988; Kovacs & Meyers, 2000; Habib & Ruff, 2008). The canalis triosseus in some flightless, diving taxa, such as modern penguins, is enlarged to allow the robust tendon of a well-developed m. supracoracoideus to pass through. Penguins acquire propulsive force from the wing-upstroke supported by the powerful m. supracoracoideus, while most flightless auks require this muscle for the recovery stroke in the water (Rayner, 1995; Johansson & Aldrin, 2002). The enlargement of the canalis triosseus in penguins is achieved by the elongation of the processus acrocoracoideus of the coracoid and the equally elongated processus acromialis claviculae (Miller & Howard, 1949; Schreiweis, 1982; Bannasch, 1994; Ksepka & Ando, 2011). The basic structure of the canal in the flightless auks is similar to that in penguins.

**Figure 2:**
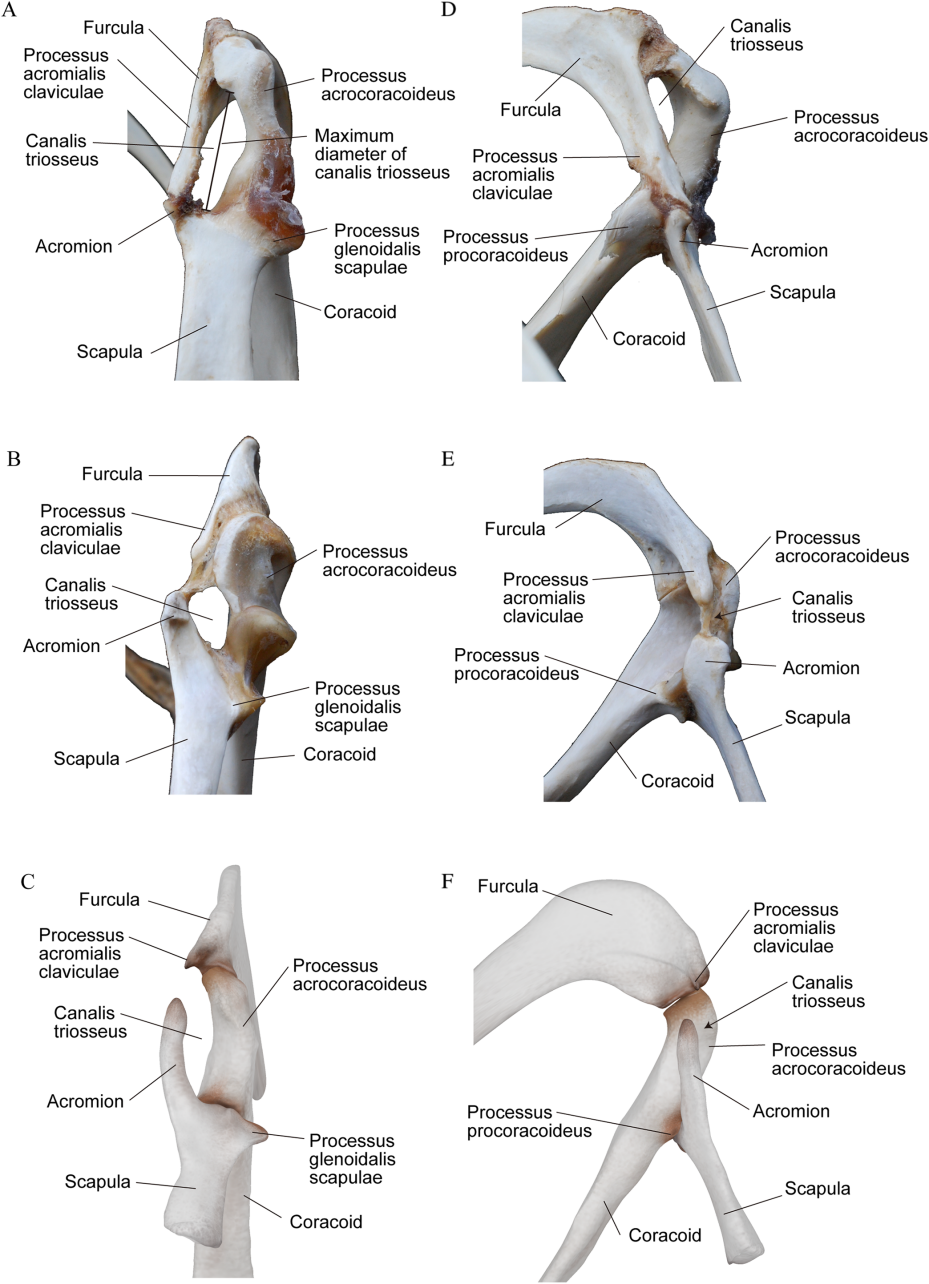
Canalis triosseus of the right shoulder girdle. (A), (D) *Pygoscelis papua* (AMP-R35). (B), (E) *Phalacrocorax capillatus* (AMP-R51). (C), (F) YM-G-100206, reconstructed by 3D computer graphic. (A)–(C) Dorsal view and (D)–(F) dorsomedial view. Not to scale.

In the past, the structure of the canalis triosseus in plotopterids was unknown because of the poor preservation of this region of the bone. The role of the m. supracoracoideus has not been discussed despite the general assumption that plotopterids performed penguin-like underwater propulsion with penguin-like wings, although the large size of the muscle could have been inferred by the elongate processus acrocoracoideus of the coracoids which would have increased the size of the triosseal canal. Here we report a well-preserved scapula of a plotopterid, YM-G-100206 (Fig. 1) from Japan (Jinnobaru Formation, latest Early Oligocene) that reveals the fine structure of the cranial scapula and enables the composite reconstruction of the canalis triosseus (Fig. 2) in plotopterids.

## Geologic Settings

YM-G-100206 was found by KF on April 29, 2013 in situ from the Jinnobaru Formation, Ashiya Group (Rupelian, latest Early Oligocene, ca. 29 Ma) at Hikoshima, Shimonoseki-City, Yamaguchi Prefecture, Japan. Hikoshima Islands in the Kanmon strait, such as Ainoshima island and Umashima island, and the northern part of Kyushu area have yielded many plotopterid specimens, including the type specimens of *C. hexeris*, *Copepteryx titan*, and other reported specimens (Hasegawa et al., 1979; Olson & Hasegawa, 1996; Kameya, Fukata & Hasegawa, 2001; Kawano & Kawano, 2001; Okazaki, 2009). Those areas comprise Oligocene strata, and invertebrate and vertebrate fossils have been found there (Tomita & Ishibashi, 1990; Okamoto & Sakai, 1995; Tomita & Oji, 2010; Okazaki, 2012; Fukata et al., 2014).

YM-G-100206 was found in the medium-grained sandstone of the Jinnobaru Formation, Ashiya Group. The Ashiya Group consists of Yamaga, Norimatsu, Jinnobaru, Honjyo, and Waita formations (Nakae et al., 1998). These five formations were originally described as three formations: Yamaga, Sakamizu, and Waita. The Norimatsu Formation was the upper part of the original Yamaga Formation, and the Jinnobaru Formation was the lowest part of the Sakamizu Formation (Nakae et al., 1998). Both fission track dating and biostratigraphic studies indicate that the lower Ashiya Group is from the Early Oligocene, while the Jinnobaru Formation was not directly dated. The fission track dating shows that the age of the bottom of the Yamaga Formation is 31.7 ± 2.3 Ma (Ozaki & Hamasaki, 1991) and that of the Norimatsu Formation is 30.3 ± 1.2 Ma (Murakami, Imaoka & Ganzawa, 1989). Okada (1992) identified that the zonal assignment for the Norimatsu and Sakamizu formations was CP19a, which ranges from the late Early Oligocene (29.9 Ma) to the early Late Oligocene (27.6 Ma) (Berggren et al., 1995). Since the Jinnobaru Formation was the lowest part of the Sakamizu Formation, we consider that the age of the Jinnobaru Formation is the latest Early Oligocene (ca. 29 Ma).

The Jinnobaru Formation in the Hikoshima Nishiyama area is mainly composed of silt-grade to coarse-grained sandstones. Several layers of rounded pebble to cobble conglomerates, which belong to the lowermost horizons, can be observed in the southern shores in this area. Beds dip NW-SE (or WNW-ESE) between 8° and 9°, and in general strike NW (Okamoto & Sakai, 1995).

## Materials and Methods

The specimen, YM-G-100206, is a well-preserved cranial portion of a right scapula (Fig. 1A–1D) housed at the Yamaguchi Museum. The original size of the scapula was estimated based on the dimensions of known plotopterid scapulae (Fig. 1). Dimensions were measured with a vernier caliper to within 0.1 mm (Table 1). A coracoid of Emperor penguin (*Aptenodytes forsteri*, USNM 346257) was digitally measured from a captured image on Illustrator CS5 software (Adobe). Anatomical terminology follows Baumel & Witmer (1993). The canalis triosseus was reconstructed as a three-dimensional computer graphic model using the scanned data of YM-G-100206 for the scapula, YM-G-100207, a plotopterid coracoid for the coracoid, and AMP 44 (*H. abashiriensis*) for the clavicle. They were scanned using the scanner function of a da Vinci AiO 3D printer (XYZ printing, Inc., San Diego, CA, USA). Then the polygon data were imported to Zbrush digital sculpting and painting program (Born Digital, Inc., Tokyo, Japan) to fill the deficits and to reconstruct the canalis triosseus. The three-dimensional models of the coracoid and clavicle were scaled to match the size of YM-G-100206. Specimens used for the comparison of the canalis triosseus (Fig. 2) are AMP-R35 (*Pygoscelis papua*) and AMP-R51 (*Phalacrocorax capillatus*).

**Table 1:** Measurements of YM-G-100206, *T. hildegardae*, *H. abashiriensis*, and *A. forsteri.*

	YM-G-100206	*T. hildegardae*	*H. abashiriensis*	*A. forsteri*
Scapula neck width (mm)	16.3	10.7	22.2	16.7
Ratio to YM-G-100206	–	0.66	1.36	1.03
Scapula length w/o acromion (mm)	167.4–185.4*	121.7	228	211.6
Ratio to neck width (mm)	–	11.38	10.27	12.67
Scapula length with acromion (mm)	200.4–215.4*	141.1	–	216.2
Maximum diameter of triosseal canal (mm)	ca. 33	ca. 19.4	–	31.4

**Notes:**

The estimated scapula lengths of YM-G-100206 are based on the ratio of scapula neck width and scapula length in *T. hildegardae* (larger values) and *H. abashiriensis* (smaller values). The maximum diameter of the triosseal canal is estimated using the length of the acromion.

* Estimated value.

## Systematic Paleontology

Aves Linnaeus, 1758
Neornithes sensu Cracraft, 1988
Plotopteridae Howard, 1969
Plotopteridae gen. et. sp. indet.
Figure 1

### Material

YM-G-100206, cranial end of right scapula.

### Locality and horizon

The specimen was collected from the medium-grained sandstone of the Jinnobaru Formation, Ashiya Group at Hikoshima in Shimonoseki-City, Yamaguchi Prefecture, southwest Japan.

### Description

The acromion of the scapula in YM-G-100206 is prominently well-developed as an elongate process, which is one of the diagnostic features for the family Plotopteridae (Olson, 1980). In addition to that, the size of YM-G-100206 indicates that it belongs to a large-sized bird (Table 1). The combination of these two features indicates that the specimen is only referable to the family Plotopteridae. YM-G-100206 is distinguishable from the scapulae of *T. hildegardae* and *H. abashiriensis* (Olson, 1980; Sakurai, Kimura & Katoh, 2008) primarily by size (Fig. 1; Table 1). When the scapula neck width is compared, *T. hildegardae* is about 66% of YM-G-100206, and *H. abashiriensis* is about 136% of YM-G-100206, excluding the possibility of it being conspecific with these species. YM-G-100206 is distinguishable from *T. hildegardae*, the only plotopterid from which the cranial end of the scapula is known, in having (1) a longer, more cranially directed acromion with a slightly swollen cranial end and an elongated depression on the ventral surface, (2) a distinct tuberculum coracoideum, (3) a shallow notch (sulcus supracoracoideus) dorsal to the tuberculum coracoideum, and (4) an origin of m. subscapularis caput mediale. The scapular size of YM-G-100206 could be equivalent to that in *C. hexeris* in which the scapular morphology is unknown. The size of forelimb elements in the holotype of *C. hexeris* are between those in *T. hildegardae* and *H. abashiriensis*. Considering this ambiguous condition, we did not name YM-G-100206, despite the diagnostic features above.

The cranial part of YM-G-100206 is almost completely preserved. It includes the acromion, tuberculum coracoideum, processus glenoidalis scapulae, and neck (Fig. 1), although the scapular blade is not preserved. The long acromion in YM-G-100206 protrudes cranially with a weak lateral curve. The cranial third slightly swells (Fig. 1), however, it does not have the articular surface for the processus acromialis claviculae (facies articularis clavicularis). The caudal two-thirds have a triangular cross section. The medial and lateral surfaces are smooth, while the ventral surface has an elongated depression. In lateral view, the ventral surface of the acromion and the shallow notch (sulcus supracoracoideus) dorsal to the tuberculum coracoideum contribute to the large canalis triosseus. The tuberculum coracoideum is a low, vaguely elliptical boss (Fig. 1). The cotyla scapularis in plotopterid coracoids is a round depression (Olson, 1980), and it must have formed the cotyla/tuberculum type of coracoscapular joint (Baumel & Witmer, 1993) with the tuberculum coracoideum similar to that of penguins. The processus glenoidalis scapulae is a rounded-triangular projection, slightly bending posteromedially (Fig. 1). The facies articularis humeralis is a triangular surface on the medial side. The neck is compressed with smooth lateral and medial surfaces. There is a tiny projection on the ventral margin of the neck, about 20 mm caudal to the angle of the processus glenoidalis scapulae. This is the origin of m. subscapularis caput mediale observed in some fossil penguins and occasionally in extant penguins (Ando, 2007).

## Results

The original scapular size of YM-G-100206 was estimated based on the ratio between the neck width and the scapular length without acromion of the scapulae in known plotopterids. The scapular length without acromion is the length from the tuberculum coracoideum to the ventral angle of the scapular blade (Fig. 2), and the dimension is available in *H. abashiriensis* and *T. hildegardae*. The ratios range from 1,027% to 1,137.5% of the neck widths of the scapulae, respectively (Table 1). The original length of YM-G-100206 is estimated at 167.4–185.4 mm, and the total scapular length could have been 200–215 mm by adding 33 mm, the length of the acromion. The scapula without acromion in plotopterids is relatively shorter than that of the Emperor Penguin (*A. forsteri*); however, the estimated scapular size in YM-G-100206 is comparable.

### Size estimates for YM-G-100206

Size differences in several skeletal elements between *H. abashiriensis* and *A. forsteri* imply that their scapular size more or less reflects the body size. The scapula of *H. abashiriensis* is about 33% larger than that of YM-G-100206 (Table 2), and several skeletal elements of *H. abashiriensis* are about 32–47% larger than those in *A. forsteri* (Table 2). These measurements could suggest that the body size of YM-G-100206 approached that of an Emperor penguin.

**Table 2:** Comparison of *H. abashiriensis* and *A. forsteri* in selected skeletal elements.

	*H. abashiriensis*	*A. forsteri*	Ratio to *A. forsteri*
Scapula neck width (mm)	22.2	16.7	1.33
Sternum width (mm)	181.6	125.0	1.45
Os coxa length (mm)	340.0	257.0	1.32
Femoral length (mm)	178.9	121.6	1.47

### Reconstruction and size of the triosseal canal in YM-G-100206

The maximum diameter of the canalis triosseus is here defined as the length from the base of the processus procoracoideus of the coracoid to the caudal margin of the processus acrocoracoideus (Figs. 2A and 2C) that occupies the lateral margin of the elongate canalis triosseus in flightless wing-propelled diving birds. In plotopterids, the length of acromion of the scapula can be a proxy for the maximum diameter of the canalis triosseus since the acromion must be the bony medial margin of the canalis triosseus (Fig. 2C). This is a rather conservative estimate because the acromion of the scapula and the processus acromialis claviculae must have been bridged by a ligament (Baumel & Raikow, 1993). There is no structure on the acromion of the scapula that may have formed a bony joint with the clavicle.

The reconstructed canalis triosseus in YM-G-100206 is an elongate oval in shape and the long axis is more or less parallel to the processus acrocoracoideus of the coracoid (Fig. 2). This condition is similar to that in penguins. The estimated maximum diameter of the canalis triosseus in YM-G-100206 is ca. 33 mm, which is close to the absolute size of the triosseal canal of the largest living penguin species, the Emperor Penguin (Table 1).

It is mandatory to have well-preserved scapula, coracoids, and clavicle from one individual to obtain the correct reconstruction and precise measurement of the canalis triosseus. However, we consider that the composite reconstruction can be a model of the canalis triosseus in plotopterids since the three skeletal elements of the canalis triosseal in plotopterids have a common structure respectively; the elongate acromion of the scapula, elongate processus acrocoracoideus of the coracoid, and vestigial processus acromialis claviculae.

## Discussion

Flightless, wing-propelled diving birds commonly possess a relatively large m. supracoracoideus compared with that of volant birds, to exert the upstroke movement in water (Schreiweis, 1982; Bannasch, 1994). The large m. supracoracoideus requires a wide and strong tendon and a large canalis triosseus for the tendon. This is widely seen in flightless, wing-propelled diving birds (Miller & Howard, 1949; Bannasch, 1994; Ksepka & Ando, 2011). In penguins, the tendon is as wide as the maximum diameter of the canalis triosseus to fill it (Fig. S1). The estimated maximum diameter of the canalis triosseus in YM-G-100206 (Table 1) is close to that of the largest living penguin species, the Emperor Penguin. The size of the reconstructed canalis triosseus suggests the wide and thick tendon for m. supracoracoideus, and, consequently, a large m. supracoracoideus. Estimation of the volume of m. supracoracoideus based only on the sizes of the acromion of the scapula and reconstructed canalis triosseus is afflicted with some uncertainty, but our data suggests that the muscle in plotopterids must have been much larger than that of suloid birds (Sulidae, Anhingidae, Phalacrocoracidae), the volant relatives of plotopterids (Olson, 1980; Mayr, 2005; Smith, 2010).

The size of m. supracoracoideus in fossil birds can be estimated by several osteological correlates in addition to the size of the canalis triosseus (Ando, 2007; Ksepka & Clarke, 2010; Smith & Clarke, 2015). In penguins and auks, the attachment area of m. supracoracoideus, which is known from the intermuscular line on the sternal body and the keel, is very wide, reflecting the large size of m. supracoracoideus. In addition, the insertion of m. supracoracoideus on the humerus in penguins and auks is elongate to accommodate the massive tendon of m. supracoracoideus. The other important muscle for the underwater flying is m. scapulohumeralis caudalis. The size of this muscle can be inferred from the posterior width of the scapular body since this region is the origin of the muscle (Schreiweis, 1982). In plotopterids, an elongate insertion of m. supracoracoideus on the humerus and a scapula with a wide scapular body has been illustrated (Olson, 1980; Sakurai, Kimura & Katoh, 2008; Dyke, Wang & Habib, 2011) while a sternum in which the intermuscular line is recognizable is not known.

A large canalis triosseus is formed by different osteological structures in flightless, wing-propelled diving birds. All of these birds have a long processus acrocoracoideus as the lateral border of the canal in common, but the medial borders are different. In penguins and flightless auks, the long processus acromialis claviculae extends caudodorsally and reaches the processus procoracoideus of the coracoids and the acromion of the scapula, almost solely forming the medial border of the canal (Fig. 2). The acromion of the scapula and the processus procoracoideus of the coracoid contribute only to the caudal margin of the canal.

The structure of the canalis triosseus in plotopterids reconstructed here is quite different from that of penguins and flightless auks. Contrary to penguins and auks, the processus acromialis claviculae is commonly vestigial in plotopterids. The process slightly protrudes beyond the facies articularis acrocoracoidea in *H. abashiriensis*, while it does not exceed the facies articularis acrocoracoidea in *C. hexeris*. The process was considered not to be preserved in *C. hexeris* (Olson & Hasegawa, 1996), but it exists as a rudimentary projection. The tiny processus acromialis claviculae cannot have contributed to the canalis triosseus in plotopterids, conversely, the long acromion of the scapula formed the medial border of the canalis triosseus.

Extant suloid birds possess characteristic morphology of the shoulder girdle region that is different from those of other birds, in the shoulder girdle region. The arcomion of suloids is well-developed. Coracoids and furculae have uniquely shaped articular surfaces (facies articularis clavicularis of the coracoids and facies articularis acrocoracoidea of the furcula), and sterna and furculae articulate as well (facies articularis furculae of the sternum and apophysis furculae) unlike in most other birds. This latter articulation has also been reported in the recently described Paleocene penguin *Sequiwaimanu* (Mayr et al., 2017). Plotopterids basically share these features but they have a more specialized structure in the canalis triosseus. The processus acromialis claviculae in suloids is longer and more distinct than that in plotopterids, but much shorter than that in penguins (Fig. 2). It exceeds the facies articularis acrocoracoidea, but never reaches the processus procoracoideus of the coracoid. The acromion of the scapula protrudes cranially, coming close to the processus acromialis claviculae, forming the medial border of the canalis triosseus. The acromion of the scapula and the processus acrocoracoideus of the coracoid are much shorter than those in plotopterids. The elongation of the acromion and specialization of the canalis triosseus in plotopterids was probably derived from the condition in the suloids. Although a sister-group relationship between penguins and plotopterids has been suggested (Mayr, 2005; Smith, 2010; Kawabe, Ando & Endo, 2014), the condition of the acromion of the scapula supports the close relationship of plotopterids and suloids. By contrast, elongation of the processus acromialis claviculae never occurred in plotopterids.

The cotyla/tuberculum type of coracoscapular joint is considered to be primitive in living birds (Baumel & Witmer, 1993); however, that type of joint is commonly observed in flightless wing-propelled diving birds, such as penguins and extinct flightless auks (Miller & Howard, 1949; Ksepka & Ando, 2011). It is uncertain if the primitive structure had a similar functional role in early birds and more advanced diving birds; the structure might be advantageous in the latter. In those birds, the pulling tension from the m. supracoracoideus is stronger than that of the volant birds and the structure might be retained to support the canalis triosseus in which the tendon of the m. supracoracoideus passes. This type of joint in plotopterids might also have contributed to making the shoulder girdle more rigid. Extant suloid birds do not have that type of joint. The combination of the suloid-like structure in the canalis triosseus and penguin-like coracoscapular joint is unique to plotopterids.

It has been hypothesized that plotopterids were penguin-like, flightless, wing-propelled diving birds, based on the penguin-like wings that can make a propulsive force, and the suggestion of the powerful upstroke muscles essential to the propulsion in water (Howard, 1969; Olson & Hasegawa, 1979), though the assessment of the swimming/diving capability in plotopterids is scarce (Howard, 1969; Olson, 1980). Howard (1969) implied that *Plotopterum joaquinensis* was a less capable swimmer than were auks and penguins, based on the fragmentary coracoid. Olson (1980), however, suggested that *T. hildegardae* was capable of producing a propulsive force using the wing-upstroke as did penguins since they had short, compressed wing elements and a wide scapular blade. The results of this study are consistent with the consensus that plotopterids were capable swimmers, and the assessment by Howard (1969) might have resulted from the rather plesiomorphic condition in *P. joaquinensis* (Mayr & Goedert, 2018). The acromion of the scapula in YM-G-100206 is relatively longer than that in *T. hildegardae,* as well as the canalis triosseus. This could suggest that the m. supracoracoideus was more powerful than that in *T. hildegardae*. Alternatively, there could be an allometric reason. The exact role of the wing-upstroke movement in plotopterids, namely the propulsive force production, or efficient recovery stroke, is still uncertain. We need further fossils that can provide more information on the powerful m. supracoracoideus and their wing movement. The variation of the shoulder girdle region, as indicated by a different shape of the caudal scapula in plotopterids (Mayr & Goedert, 2016), suggests that there was a morpho-functional variation in their evolutionary history. As the competition with marine mammals has been suggested as a cause of the demise of giant forms of penguins and plotopterids (Olson & Hasegawa, 1979; Ando, 2007; Ando & Fordyce, 2014), the morpho-functional variation in plotopterids could have related to such event.

Plotopterids and penguins are considered examples of evolutionary convergence (Olson & Hasegawa, 1979), and shared specialized wing elements and powerful upstroke muscles. The large canalis triosseus and a rigid coracoscapular joint in plotopterids and penguins are another example of the convergence resulting from the same functional requirement. Plotopterids are, however, distinguished from penguins in that their triosseal canal was derived from the condition in the phylogenetically close suloid birds. Both the functional requirement and the phylogenetic constraint (Reify, Thomas & Fischer, 1985) formed the structure of the canalis triosseus in plotopterids.

## Conclusion

We described one of the best preserved scapulae of a plotopterid (YM-G-100206) from the Early Oligocene Jinnobaru Formation (ca. 29 Ma) in southwest Japan. The scapula has an elongate acromion that covers the medial border of the canalis triosseus, and the elliptical tuberculum coracoideum that can accommodate the depressed cotyla scapularis in the coracoid of plotopterids. The fine structure of the scapula enables the composite reconstruction of the canalis triosseus in plotopterids. The reconstructed canalis triosseus is comparable to that in penguins in its size and shape, suggesting that the m. supracoracoideus in plotopterids was well-developed. However, the structure of the canalis triosseus was different from that in penguins. The medial border of the canalis triosseus of plotopterids was composed of the elongated acromion of the scapula, while penguins have a long processus acromialis claviculae. The condition in plotopterids was probably derived from the condition in the closely related suloid birds.

## Supplemental Information

10.7717/peerj.5391/supp-1 Supplemental Information 1 Triosseal canal with soft tissues in Yellow-eyed Penguin. The canalis triosseus is hidden by soft tissues. Unnumbered specimen (Geology Museum, University of Otago, New Zealand). Not to scale.

## Institutional Abbreviations

AMP: Ashoro Museum of Paleontology (Ashoro, Japan)
KMNH: Kitakyushu Museum of Natural History (Kitakyushu, Japan)
USNM: National Museum of Natural History (Washington, D.C.)
YM: Yamaguchi Museum (Shimonoseki-City, Japan)

## Additional Information and Declarations

### Data Availability

The following information was supplied regarding data availability: The described specimen YM-G-100206 is housed at the Yamaguchi Museum (Shimonoseki-City, Yamaguchi Prefecture, Japan).
