# Supplementary figures and images for: A well-preserved partial scapula from Japan and the reconstruction of the triosseal canal of plotopterids
Source: PeerJ. 2018 Aug 25;6:e5391. doi: 10.7717/peerj.5391 (PMC6112113; doi:10.7717/peerj.5391)

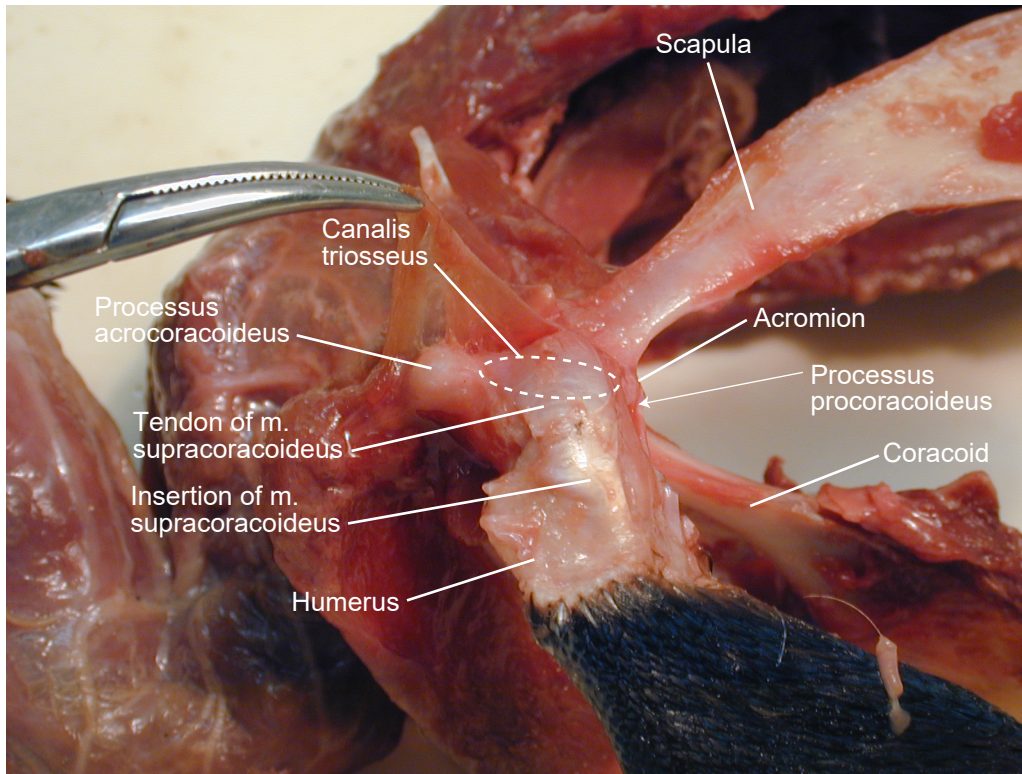

Supplement: Supplemental Information 1 — The canalis triosseus is hidden by soft tissues. Unnumbered specimen (Geology Museum, University of Otago, New Zealand). Not to scale. [file peerj-06-5391-s001.pdf]
